# Rectal Clear Cell Carcinoma Arising from Endometriosis: Case Report and Literature Review

**DOI:** 10.3390/diagnostics15151936

**Published:** 2025-07-31

**Authors:** Adriana Ioana Gaia-Oltean, Dan Boitor-Borza, Voicu Caius Simedrea, Vlad Braicu, Laura-Ancuta Pop, Romeo Micu

**Affiliations:** 1 Regina Maria Hospital, 400117 Cluj-Napoca, Romaniaromeo.micu@umfcluj.ro (R.M.); 2 “Iuliu Hatieganu” University of Medicine and Pharmacy, 400012 Cluj-Napoca, Romania; laura.ancuta.pop@gmail.com; 3 Department of Obstetrics and Gynecology, “Iuliu Hatieganu” University of Medicine and Pharmacy, 400012 Cluj-Napoca, Romania; 4 Regina Maria Hospital, Endoinstitut, 300645 Timisoara, Romania; voicusimedrea@gmail.com; 5 2nd Surgery Clinic of the County Hospital Timisoara, “Victor Babes” University of Medicine and Pharmacy, 300041 Timisoara, Romania; braicu.vlad@umft.ro; 6 Genomics Department, MEDFUTURE-Institute for Biomedical Research, 400012 Cluj-Napoca, Romania

**Keywords:** rectal endometriosis, malignant transformation, Müllerian clear cell carcinoma, clinical outcome

## Abstract

**Background and Clinical Significance:** Endometriosis is a common gynecological disease that can occasionally be associated with malignant transformation. The most common site of malignant transformation is the ovary, but there can also be rare extragonadal endometriosis-associated malignancy sites, such as the intestines, rectovaginal septum, and abdominal wall. A low number of malignant degenerations of rectal endometriosis are described in the literature. However, the majority of these cases report endometrioid adenocarcinoma as the most frequent histopathological type of tumor. On the other hand, Müllerian clear cell carcinoma is sporadic. **Case Presentation:** We present the case of a 43-year-old woman with clear cell carcinoma of the rectum, which developed on an endometriosis nodule, and the surgical outcome. Imaging of the case was performed by MRI. The patient was offered curative surgery. The pathology report confirmed a clear cell carcinoma developed on an endometriosis lesion, and immunochemistry helped in the characterization of the tumor. The patient developed a rectovaginal fistula. An ileostomy and surgical repair of the fistulous opening were performed, with a favorable postoperative recovery. **Conclusions:** Malignant transformation of endometriosis lesions is possible and should be taken into consideration. Müllerian clear cell carcinoma development within rectovaginal endometriosis is extremely rare.

## 1. Introduction

Endometriosis is characterized by the presence of endometrium-like epithelium and/or stroma outside the endometrium and myometrium, usually with an associated inflammatory process [1]. It is recognized as one of the most common chronic, estrogen-dependent gynecological diseases [2]. As an estimate, the prevalence of endometriosis is between 10% and 18% among women of childbearing age [2,3,4]. Endometriosis shows rare malignant transformation into endometriosis-associated neoplasms, predominantly clear cell carcinoma (CCC) and endometrioid adenocarcinoma. While ovarian CCC is well-documented, extraovarian cases, especially rectal CCC, are exceedingly rare and pose diagnostic and therapeutic challenges.

Bowel involvement by deep endometriosis constitutes a major challenge for the clinician and has been estimated to occur in 5–12% of patients with endometriosis [5]. Recto-sigmoid localization occupies 80–90% of the forms of digestive endometriosis [6]. The symptoms of digestive endometriosis are represented by pain (pelvic chronic pain, dysmenorrhea, dyspareunia) and digestive signs (dyskinesia, constipation), all with a cycle character and a peak during menstruation [6].

Although endometriosis is associated with a higher risk of ovarian, breast and thyroid cancers in particular, the increase in absolute risk compared with women in the general population is low [7]. Particularly, malignant transformation of colorectal endometriosis is rare, accounting for 0.25% [8]. The malignant transformation of gastrointestinal endometriosis is known as endometriosis-associated intestinal tumors (EAITs) [9,10]. The criteria for rectal endometriosis-associated cancer include the presence of pathologically confirmed endometriosis at the rectal site and the presence of pathologically confirmed malignant rectal pathology (CCC, in our case) involving the site of endometriosis.

The number of cases of malignant degeneration of rectal endometriosis described in the literature is very low. However, the majority of these cases describe endometrioid adenocarcinoma as the most frequent histopathological type of tumor. CCC development within intestinal endometriosis is rare. The Müllerian type (mCCC) is one of the two types of CCC of the colon, besides the intestinal type (iCCC) [11]. mCCC has distinct clinical, pathological, diagnostic, and prognostic aspects [12]. The differential diagnosis is represented mainly by secondary metastases of ovarian or renal origin [11]. One clinical feature of the reported mCCC is that they appear mostly in the rectum (77% of cases) or sigmoid [12]. There are very few case reports in the literature [13]. The treatment is based on surgery, possibly on chemotherapy [11], but overall it is not yet consensual.

We report a case of Müllerian rectal clear cell carcinoma arising from endometriosis and a literature review on the subject.

## 2. Detailed Case Description

A 43-year-old woman was referred for surgical treatment of rectovaginal endometriosis. Five years earlier, she underwent surgery for bilateral ovarian endometrioma, which included a right adnexectomy and a left partial ovariectomy. Three years later, the patient was diagnosed with asymptomatic endometriosis, Enzian A3 B1/1 C3, and received hormone therapy until breast cancer was diagnosed. The patient had conservative breast surgery, sentinel lymph node removal, radiotherapy, and treatment with GnRH analogs and aromatase inhibitors.

**Figure 1:**
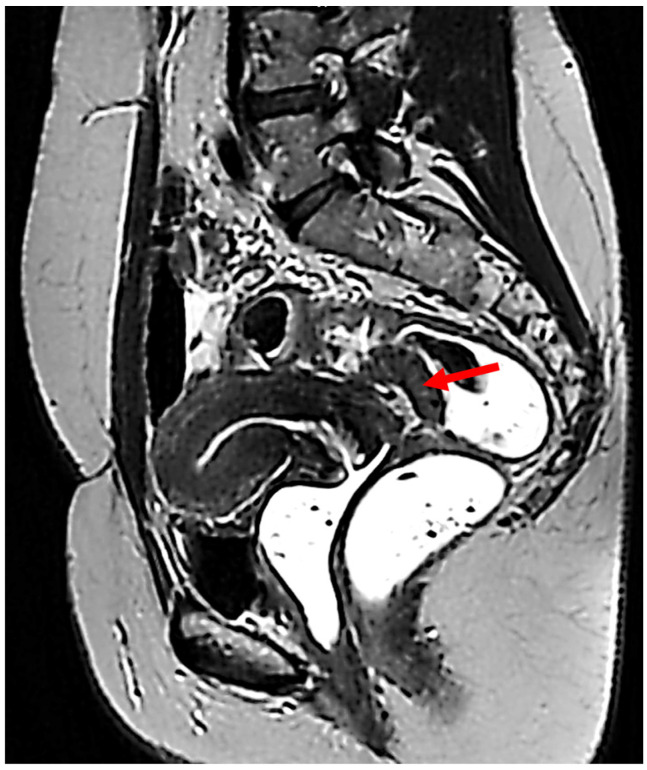
Sagittal MRI image, T2-wi sequence. A nodular lesion of 30 mm by 13 mm is depicted in the posterior vaginal fornix, infiltrating the cervix and the anterior wall of the rectum (arrow).

A transvaginal ultrasound revealed a tumoral mass of 31 mm diameter in the posterior vaginal fornix, with an intense Doppler signal. MRI showed deep endometrial lesions in stage 4rASF, Enzian T2/x, A3, B2/2, C3, FA. Furthermore, a 30 mm-by-13 mm nodular lesion with a 10 mm-by-12 mm portion presenting slight hyperintense signal in T1 FatSat sequence was discovered in the posterior vaginal fornix (arrow in Figure 1). This lesion infiltrated the posterior aspect of the cervix, uterine torus, uterosacral ligaments, posterior vaginal fornix, superior portion of the rectovaginal septum, and the anterior rectal wall. The suspicious MRI findings prompted a biopsy of the lesion, which revealed high-grade clear cell carcinoma.

**Figure 2:**
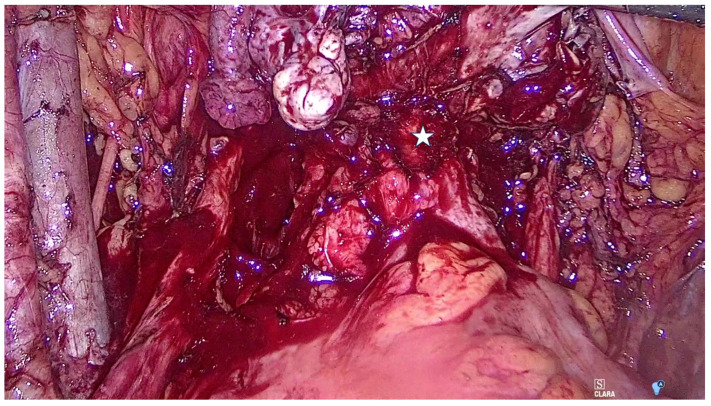
Image captured during laparoscopy. The endometriotic nodule in the rectovaginal septum is indicated by the star. The pathology report confirmed a clear cell carcinoma developed on an endometriosis lesion, and immunochemistry helped in the characterization of the tumor.

The patient was scheduled for curative surgery, which included bilateral ilio-hypogastric and obturator lymphadenectomy, total laparoscopic enlarged hysterectomy Piver 2 with left adnexectomy, identification of the recto-vaginal nodule (star in Figure 2), rectal stapling at four cm above the external rectal orifice, followed by transvaginal uterine and rectum extraction. Resection of the rectum above the nodule and mechanical termino-terminal anastomosis were carried out. Sampling of two right hypogastric lymph nodes, and four left hypogastric and left obturator lymph nodes was performed. The colonoscopy showed no leakage from the rectal suture. The patient left the hospital five days after surgery.

**Figure 3:**
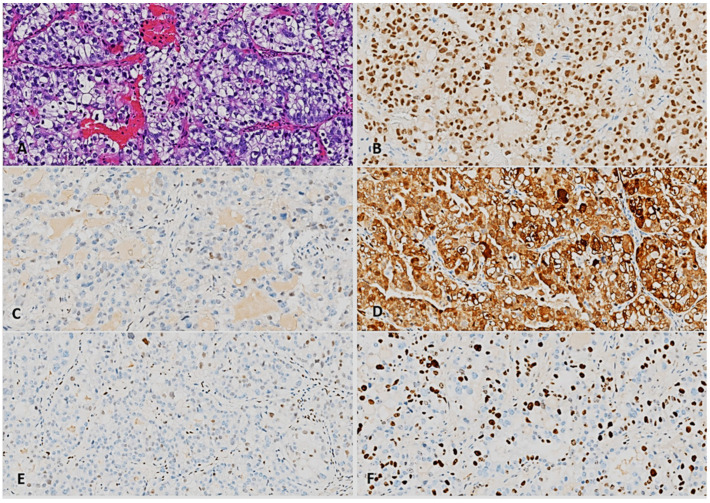
Morphological and immunohistochemical features of the tumor: (**A**) large tumor nodules consisting of large cells, pleomorphic nuclei, frequently clear cytoplasm and relatively low mitotic activity (HE stain, 200×); (**B**) diffuse, intense PAX8 positivity in tumor nuclei (200×); (**C**) rare nuclei with low *p53* expression, indicating a wild-type *TP53* gene (200×); (**D**) combined p16 expression, mostly cytoplasmic, but also nuclear, of predominantly high intensity in tumor cells (200×); (**E**) low-intensity estrogen receptor expression in rare tumor cells (200×); (**F**) moderate proliferating activity in tumor cells (Ki67, 200×).

The pathology report confirmed a clear cell carcinoma developed on an endometriosis lesion, and immunochemistry helped in the characterization of the tumor (Figure 3). The histopatological exam of the right and left hypogastric lymph nodes, and left obturator lymph nodes showed no signs of malignancy. Additionally, a histological examination of eight perirectal and two paracervical lymph nodes revealed normal findings. There were no signs of angiolymphatic or perineural invasion. The procedure was therefore considered as a R0 resection.

The patient was readmitted to the hospital two weeks after for feces evacuation through the vagina. She had a colonoscopy, which revealed a rectovaginal fistula. An ileostomy and surgical repair of the fistulous opening were performed. The patient was released from the hospital two days after surgery and had stoma closure after three months, with a favorable postoperative recovery. Due to the limited disease, the patient did not require adjuvant chemotherapy. She is doing well at present.

Ethical approval for this retrospective case was compliant with the ethical standards of the institutional research committee, according to the Helsinki Declaration. The consent for the publication of photos was obtained, ensuring that the patient could not be identified from the images.

## 3. Discussion

Cases of extra-ovarian CCC development within rectovaginal endometriosis are extremely rare and we were able to find no more than six cases in the literature. In Table 1, their clinical features are presented.

Given that our patient was asymptomatic, an early diagnosis of cancer was not possible. Recent research identified two cases of concurrent endometriosis and rectal endometrioid carcinoma among 1397 women [19], but no CCC arising within rectal endometriosis.

The mCCC is found to be rather indolent [12]. It arises in intestinal endometriosis, and it is associated with Müllerian duct remnants. mCCC has distinct clinical, pathological, diagnostic, and prognostic aspects, as it appears especially in the rectum (77% of cases) or sigmoid [12].

In our case, the potential of malignancy was suggested on MRI, which is the most sensitive imaging method for the assessment of rectal wall involvement. The lesion’s location in the posterior vaginal fornix made the biopsy easier.

Given the benefits of laparoscopy or robotic surgery for the treatment of endometriosis and endometriosis-related diseases, minimally invasive surgery was the best option in our case. Postoperative complications such as rectovaginal fistula were anticipated, given the complexity of the procedure and the characteristics of the infiltrated and inflamed tissue. Even in experienced hands, infiltration of the vagina triples the risk of developing a bowel fistula [20].

Endometriosis, one of the most frequent gynecological pathologies, is reported with a significantly growing prevalence among women of childbearing age [3]. The risk of malignant transformation of endometriosis is increased in perimenopause and postmenopause [13]. Malignant transformation of gastrointestinal endometriosis is described in the literature, even though it is rare [9]. The most frequent site of the malignant transformations is the ovary, but other extra-ovarian sites were also been reported. Even though endometriosis turning malignant is extremely rare, it must be considered. Endometriotic lesions, irrespective of subtype, if left intact, would generate cancer-associated mutations as part of replicative aging, oxidative stress and perhaps other factors yet to be identified and, in some rare cases, develop cancer [21]. 

The most common histopathological type of extraovarian endometriosis-associated tumors is endometroid carcinoma, with a rate of up to 90%, and only 5% are classified as CCC type [9]. CCC development within rectovaginal endometriosis is extremely rare. The mCCC has been identified in women [11].

In the most common localization of EAITs, the rectosigmoid colon, the patients report symptoms such as pelvic/abdominal pain, gastrointestinal bleeding, bowel obstructions, and pelvic mass [10,14].

In our case, the differential diagnosis included endometrioid carcinoma, cervical cancer, and metastatic rectal CCC. The presence of adjacent endometriosis, as well as a history of endometriosis, strongly indicates primary rectal CCC [12]. The histological diagnosis of clear cell cancer can be difficult, which may lead to the diagnosis of unknown or undifferentiated histology thus causing underestimation of true number of cases [22]. The value of biomarkers in the diagnosis of deep endometriosis is unsignificant [23].

Immunohistochemistry assists in demonstrating the Müllerian origin (CK7, CK20, CDX2, intense PAX8 positivity in tumor nuclei) and warranted the establishment of a correct diagnosis [10,14]. CCC immunophenotype is specifically characterized by the expression of hepatocyte nuclear factor 1-beta (HNF-1β), negative staining for WT1, estrogen-receptor (ER), and progestin-receptor (PR) expression, and wild-type pattern of *p53* expression [24]. Unfortunately, in our case, the immunohistochemical stains for HNF-1β and WT1 were not carried out, but the genital origin was confirmed by the PAX8 staining. Meanwhile, the expression of p16 and Ki67 excludes a G3 endometrioid adenocarcinoma, with occasional cytoplasmic clarifications. The positivity of p16 and Ki67 and the presence of rare nuclei with low *p53* (wild-type) expression served as a differential diagnostic criterion to rule out cervical high-grade serous carcinoma. The focal, low-intensity ER expression in rare tumor cells indicated that the tumor was not estrogen-dependent, as would be the case with an endometrioid carcinoma.

The therapeutic decision is based especially on imagistic investigation and biopsy with histopathological results. These contribute to the surgical indication and type of surgery, and in some cases to adjuvant treatment [10,14]. Intestinal segmental resection with additional total hysterectomy, bilateral salpingooophorectomy, and omentectomy were performed in our case. Lochner et al. reported the first case of rectum mCCC arising from endometriosis, in which case bilateral pelvic and para-aortic lymph node dissection and, with mesorectal lymph node metastasis were performed [14]. Surgical resection remains the cornerstone of treatment, but optimal adjuvant therapy is unclear. In our case, the development of clear cell carcinoma on an endometriotic nodule in the rectal wall rather than in the ovary or endometrium, for which staging protocols are available, restricts the use of pTNM staging. However, the lymph nodes and the resection margins of hysterectomy and rectal specimens showed no signs of malignancies.

The pathophysiology of malignancies linked to endometriosis has been questioned by a variety of theories over time. Tumorigenesis is believed to be promoted by hormone imbalance, inflammatory response, and reactive oxygen species (ROS) [13]. Genetic perspectives, however, are given preference in present times. The genetic link between endometriosis and cancer is highly debated, with a focus on identifying pathogenetic mutations that occur in cells during the development of the disease. Current genetic theories that could explain the pathogenesis of endometriosis-associated cancers include the implications of specific miRNAs, the pattern of loss of heterozygosity, the molecular mechanism involving gene mutations, and microsatellite instability. From the molecular point of view, endometriosis is characterized by a broad and significant genetic variety, which can lead to a wide genetic instability. Histologically benign endometriosis can hide molecular abnormalities, which in time can lead to a malignant transformation [25]. Extensive genetic investigation, including next-generation sequencing, has explored the role of molecular factors in the development of endometriosis in malignant lesions by researching their interrelated ways. In deep ectopic lesions, detected variants were significantly more often located in cancer driver genes, whereas in eutopic endometrium, there was no such distribution [26]. In some cases, somatic tumor testing pointed out mutations in *ARID1A, PTEN* and *p53*, which are tumor suppressor genes associated with microsatellite instability [17,24]. *PIK3CA* mutations are common in CCC, while *KRAS* mutations (10%), *TP53* mutations (<10%), and mismatch repair deficiency (0–6%) are uncommon [27]. Genetic instability is associated with both ovarian endometriosis and ovarian cancer, according to studies [28]. This is also discussed in deep endometriosis but has not been well researched [29]. In endometriosis, the frequency of an aberrant somatic gene mutation appears to be higher, as one-quarter of patients with deep endometriosis exhibit frequent gain-of-function mutations in the *KRAS*, *PIK3CA*, and *PPP2R1A* genes, as well as loss-of-function alterations in *ARID1A* [29]. Both ovarian endometriosis and ovarian cancer share common altered genes in terms of mutation or expression, including *ARID1a*, *KRAS*, *PIK3CA*, *PTEN*, *TP53*, *CTNNB1* [28,29]. Somatic mutations of the *ARID1A*, *PIK3CA*, *KRAS*, and *PPP2R1A* genes were also detected in the majority of benign deep endometriosis lesions, including colorectal endometriosis [30]. For instance, *ARID1A*, a tumor suppressor gene with a 46% mutation rate in ovarian clear cell carcinomas, was also found in 10% of colon cancer cases [16].

In the future, understanding the relationship between genital and extragenital endometriosis, as well as gene expression and polymorphism, may contribute to a novel approach to endometriosis management, focusing on new therapeutic options, namely personalized therapy. Mutation signature-based whole-exome sequencing could be useful to select an adjuvant chemotherapy regimen [31]. Nevertheless, according to ESHRE guideline form 2022, genetic testing in women with suspected or confirmed endometriosis should only be performed within a research setting [32].

Epigenetics has a critical role in endometriosis pathophysiology. miRNAs regulate gene expression and have crucial consequences for carcinogenesis through their up and down regulation. Transcriptional microarray data have shown that miRNA expression levels varied between tumor and normal tissues [33]. They modulate gene expression, and growing evidence supports their potential as biomarkers in endometriosis. miRNAs, including miR-200, miR-17, miR-34, miR-1, and miR-155, have been shown to play a molecular impact on endometriosis [28]. DNA methylation is a well-studied epigenetic process that involves changes in the enzymes that methylate and demethylate DNA [34]. Abnormal DNA methylation alters the turnover of endometriotic lesions [29]. Histone dysregulation is also considered an epigenetic alteration. Lipopolysaccharide (LPS) promotes histone lactoylation and facilitates colon cancer cell migration [35]. Epigenetic alterations, however, are reversible. As a result, there is potential for advancements in therapeutic epigenetic mechanisms that may influence endometriosis progression.

The staging of rectal CCC is challenging because there is no guideline on how to consider it as a primary colon carcinoma or as a primary peritoneal carcinoma [36]. The distinction between mCCC and colorectal carcinoma should impact the treatment and follow-up. The surgical treatment is almost similar, considering that they both arise within the colonic wall, although not both within the colonic mucosa. This distinction between mCCC and colorectal carcinoma is typically straightforward using immunohistochemistry. Immunostains include CK7 and CK20, and sometimes also tissue-specific markers such as CDx-2 and Pax-8. Prudence is advised while considering hormone therapy, as the ontogenesis of cancer from endometriosis remains insufficiently comprehended.

This work provides three important practical recommendations. To begin, while rectal mCCC caused by endometriosis is uncommon, it should be considered, particularly in women with long-standing endometriosis and rectal symptoms. Second, the diagnosis is based on clinical symptoms, imagistic investigation, histological data, and immunohistochemistry, the latter two of which are critical for establishing the diagnosis and distinguishing between primary colon cancer and peritoneal carcinoma. Third, surgery is the primary choice for treating this pathology.

## 4. Conclusions

Rectal mCCC arising from endometriosis is a rare, aggressive malignancy requiring multidisciplinary care. It should be considered in women with long-standing endometriosis and rectal symptoms. Prudence is advised while considering hormone therapy, as the ontogenesis of cancer from endometriosis remains insufficiently comprehended. Immunohistochemistry is critical for differentiating it from primary colorectal adenocarcinoma. Early diagnosis and radical surgical resection remain the cornerstones of treatment which may improve outcomes, but evidence remains limited and optimal adjuvant therapy is unclear. Further research is warranted to establish standardized guidelines. Molecular studies to identify targeted therapies (e.g., *ARID1A* mutations) are necessary.

## Figures and Tables

**Table 1:** Review of cases with clinical characteristics.

No.	First Author (Year)	Age of the Patient (Years)	Symptoms	Investigations	Type of Surgery
1	McCluggage [14] (2001)	65	Abdominal pain Hematochezia mucus stool	Barium enema	Segmental resection
2	Pokieser [15] (2003)	40	Lumbosacral pain loss weigh night sweats intermittent fever	CT scan	Palliative deep rectum resection Liver biopsy
3	Finkelstein [10] (2010)	41	Hematochezia Diarrhea	Colonoscopy Biopsy	Segmental resection
4	Kyueng Wan Min [16] (2012)	51	Hematochezia	CT scan Endoscopy Biopsy	Low anterior resection Complementary surgery (hysterectomy, bilateral salpingo-oophorectomy, omentectomy)
5	Yu Okazawa [17] (2014)	83	Hematochezia	Barium enema CT scan MRI Colonoscopy Biopsy CEA, CA125, CA 19-19-VN	Low anterior resection
6	Lochner [18] (2025)	68	Hematochezia Mucus stool	CT scan MRI Endoscopy Biopsy CEA, CA125-VN	Proctosigmoidectomy Omentectomy Modified radical hysterectomy with bilateral salpingo-oophorectomy, peritoneal biopsy, bilateral pelvic and para-aortic lymphadenectomy

## Data Availability

The original contributions presented in this study are included in the article. Further inquiries can be directed to the corresponding author.

## Abbreviations

The following abbreviations are used in this manuscript:

mCCC	Müllerian clear cell carcinoma
CCC	Clear cell carcinoma
EAIT	Endometriosis-associated intestinal tumor
iCCC	Intestinal clear cell carcinoma
